# A Polygon-Based Joint Feature Construction and Matching Algorithm for Star Identification in Star Sensors

**DOI:** 10.3390/s26196013

**Published:** 2026-09-23

**Authors:** Lingfeng Liang, Hanwei Zhang, Changhua Liu

**Affiliations:** 1 School of Surveying and Land Information Engineering, Henan Polytechnic University, Jiaozuo 454003, China; lchnj@163.com; 2 School of Civil Engineering and Geomatics, Shandong University of Technology, Zibo 255000, China; zhanwei800@163.com

**Keywords:** star sensor, star identification, polygon matching, multi-feature fusion

## Abstract

**Highlights:**

**What are the main findings?**

- Fusing inter-star angular distances, neighboring-edge angles, relative magnitude differences, and multi-order similarity invariants into a single statistically weighted polygon descriptor achieved the highest identification rate of all tested algorithms in each of the eleven degraded-data environments (84.14–92.74%), remaining above 92% under 1.0 px centroid noise or 20% false stars.
- The five-star-first, four-star-fallback hierarchical retrieval kept the 95th-percentile solving time sub-second with zero timeouts in every environment; under the interference-free condition, typical (P50) solving time was 20.5% lower than the triangle algorithm, 86.3% lower than the pyramid algorithm, and 80.6% lower than the geometric polygon algorithm.

**What are the implications of the main findings?**

- High-dimensional joint features are evaluated using the covariance-based Mahalanobis distance, with the decision threshold determined according to the chi-square distribution. This approach effectively reduces candidate-matching ambiguity encountered by conventional low-dimensional geometric methods under centroid noise, false-star interference, and compound disturbances. Combined with an optimized retrieval strategy that prioritizes five-star polygons and uses four-star polygons as a fallback, the proposed method achieves a balance between identification reliability and real-time performance, providing a practical approach for autonomous lost-in-space attitude acquisition using onboard star sensors.

**Abstract:**

To address the insufficient discriminative capability of features and the susceptibility to mismatches under noise in conventional geometric-feature-based star identification algorithms, this paper proposes a polygon-based star identification algorithm that integrates multiple features. The algorithm combines inter-star angular distances, angles between adjacent edges, relative stellar magnitude differences, and multi-order similarity invariants to construct joint polygon features. The Mahalanobis distance is used for matching decisions, thresholds are determined from the chi-square distribution, and an optimized retrieval strategy prioritizing five-star polygons and supplementing them with four-star polygons is designed. Using 372 measured star images as the basis for 11 test conditions, the proposed algorithm is compared with the triangle, pyramid, and conventional geometric polygon algorithms on a frame-by-frame basis, with each algorithm completing 4092 frame-wise solutions. The results show that the proposed algorithm achieves the highest identification rate among the four algorithms under all non-photometric perturbation conditions, ranging from 84.14% to 92.74%; its identification rates under 1.0 px star position noise and 20% star dropout are 92.20% and 86.02%, respectively, both exceeding those of the three comparison algorithms. Under all-sky lost-in-space conditions, the 95th percentile of the runtime for correctly identified frames is below 1 s in every environment, and the mean solution runtime is reduced by 41.5%, 94.6%, and 82.1%, respectively, relative to the three algorithms above. It exhibits good noise tolerance and identification efficiency and can provide methodological support for all-sky lost-in-space star identification.

## 1. Introduction

In space missions such as Earth observation and deep-space exploration, star sensors serve as core optical sensors for spacecraft attitude determination owing to their arcsecond-level measurement accuracy; all-sky lost-in-space (LIS) identification, which matches stellar identities using only a single star image without any prior attitude information, is a key manifestation of star sensor autonomy. Under in-orbit conditions, centroid extraction errors induced by high dynamics [1], increased noise and obscured star spots caused by space radiation damage [2], and false and missing stars under strong backgrounds [3] all alter the local structure of observed star images; erroneous matches propagate incorrect inertial references to attitude determination, with consequences more severe than a single identification failure. Therefore, constructing star image features and corresponding decision mechanisms that combine discriminative capability, noise robustness, statistical interpretability, and retrieval efficiency is a central issue in improving the reliability of all-sky identification.

Classical methods mainly construct identification patterns using rotation-invariant geometric quantities such as inter-star angular distances. The triangle algorithm proposed by Liebe [4] constructs a three-star pattern from a reference star and its two nearest neighbors and performs matching using inter-star angular distances and included angles, representing this class of methods, but it is susceptible to redundant matches under star position noise and requires substantial feature database storage; the pyramid algorithm proposed by Mortari et al. [5] progressively verifies candidate matches through the closed-loop consistency of four-star angular distances and remains one of the widely used benchmark algorithms; Sun et al. [6] construct dynamic angle features from angles between neighboring stars and star-pair distances and perform identification using matching scores, reducing dependence on the selection of the initial star. Chng et al. [7] formulate star identification as a global search problem in rotation space and use branch-and-bound optimization to achieve heuristic-free optimal alignment. Although these methods continually improve the reliability of geometric matching, their discriminative information remains concentrated in a small number of low-dimensional geometric quantities. When stars are densely distributed in the field of view or similar configurations occur in different sky regions, increasing the matching tolerance readily admits multiple catalog patterns into the candidate set, whereas reducing the tolerance decreases the algorithm’s tolerance to observational noise. Although global search can improve matching reliability, its computational cost is high, making it difficult to balance identification rate, mismatch control, and onboard real-time processing requirements.

To improve the description of the overall configuration of a local sky region, researchers have further extended identification patterns to polygons. Hernández et al. [8] map a polygon consisting of a reference star and its neighboring stars to complex-valued invariants that are insensitive to similarity transformations such as rotation, translation, and scaling and use these invariants to construct catalog indices. Building on this approach, Ramos-Alcaraz et al. [9] introduce a k-d tree to accelerate invariant retrieval and combine inscribed polygons with a voting mechanism to verify candidates, improving retrieval efficiency. However, the final decision still relies primarily on geometric consistency and is relatively sensitive to false and missing stars. More importantly, compressing a polygon containing rich structural information into a single or a small number of low-dimensional invariants loses some of the information that distinguishes different configurations. Under noise, the perturbation ranges of invariants from different polygons may overlap, and similar configurations may readily yield similar feature values. Therefore, low-dimensional invariants are better suited to rapid indexing, whereas relying solely on them for the final decision makes it difficult to fully exploit the complete geometric information contained in a polygon.

In addition to feature design, candidate retrieval efficiency and matching decision methods directly affect star identification performance. Wang and Xu [10] select navigation stars using random optical-axis vectors and reduce the search burden by decreasing the size of the navigation star database; Liu et al. [11] achieve rapid matching of sequential star images for precisely tracking telescopes; Kim [12] uses statistical models to describe star patterns, providing a new approach to probabilistic star identification. Overall, existing studies devote considerable attention to feature representation and candidate search efficiency, while matching decisions still frequently rely on fixed tolerances or the Euclidean distance. When features with different physical dimensions and measurement precisions, such as inter-star angular distances, included angles, magnitude differences, and complex-valued invariants, jointly contribute to a decision, simple distance metrics tend to make their contributions depend more on numerical scales than on their actual reliability. Therefore, rapid retrieval cannot replace reliable candidate decisions. Further research is needed on constructing a joint error model for heterogeneous geometric, photometric, and invariant features and forming a unified decision at a specified false-alarm probability. The Mahalanobis distance provides an appropriate statistical decision tool for this purpose: the covariance matrix enables scale normalization of feature residuals with different physical dimensions and precisions while accounting for inter-feature correlations; when the corresponding statistical assumptions hold, its square can be described by a chi-square distribution, allowing the acceptance threshold to be set from the correct-match residual model and a specified decision parameter.

Based on the above analysis, this paper uses a star polygon comprising a reference star and its neighboring stars as the basic identification unit and proposes an all-sky star identification method that integrates geometric features, relative photometric features, and multi-order similarity invariants. For feature construction, inter-star angular distances, angles between adjacent edges in the tangent plane, relative stellar magnitude differences, and the real and imaginary parts of multi-order complex-valued similarity invariants are assembled into a real-valued joint feature vector to improve the description of polygon configurations and the discrimination between candidate patterns. For matching decisions, error models are established for the three feature types separately, the statistical structure of the joint features is described using a block-diagonal covariance matrix, and a matching criterion is constructed from the joint Mahalanobis distance, with the acceptance threshold set from the chi-square distribution and a specified decision parameter under an approximately Gaussian error model; each covariance block is obtained by estimating measurement uncertainties from the local background and star-spot fitting in the original star images and then propagating the errors. For candidate retrieval, a hierarchical procedure combining “coarse screening with a k-d tree in invariant space + fine discrimination using joint features” is adopted, prioritizing five-star polygons to reduce the candidate space, falling back to a four-star index when the five-star pattern cannot provide reliable identification, and using the fifth star for consistency verification. Eleven test environments are constructed from 372 measured star images for frame-by-frame comparison with the classical triangle and pyramid algorithms and the geometric polygon algorithm. The results show that the proposed algorithm achieves a high identification rate and good computational efficiency across the test environments, providing an adjustable and extensible implementation for all-sky star identification under complex noise and different feature measurement precisions.

## 2. Joint Multi-Feature Construction

### 2.1. Definition of the Three Types of Feature Vectors

Consider a star polygon consisting of *N* stars, namely a reference star (central star) and its *N* − 1 neighboring stars. In the image-plane coordinate system, the centroid coordinates and observed stellar magnitude of the k-th star are denoted, respectively, as:

(1) $$(x_{k},y_{k}),\;m_{k},\;k=1,\ldots ,N$$

where

$x_{k},y_{k}$ denotes the centroid position of the k-th star in the image-plane coordinate system (in pixels or normalized coordinates);

$m_{k}$ denotes the corresponding observed stellar magnitude;

N denotes the number of vertices in the polygon.

Let star 1 be the reference star, with its direction vector on the unit celestial sphere denoted by $r_{1}$, and let the direction vectors of the remaining stars, $r_{k}$, be obtained by back-projection using the imaging geometry. The nonparallel direction correspondences of multiple identified stars jointly constrain the attitude, thereby eliminating the rotational ambiguity about the direction of a single line of sight.

(2) $$r_{k}\in {\mathbb{R}}^{3},\;\|r_{k}\|=1,\;k=1,\ldots ,N$$

where

$r_{k}$ denotes the unit line-of-sight vector of the k-th star in the star sensor coordinate system; the observed stellar directions are determined by the image-plane positions of the stars, and different stars in the same star image correspond to different directions.

$\|r_{k}\|=1$ denotes the unit-length constraint, which facilitates the calculation of geometric quantities such as angular distances and angles.

To make the features of observed and catalog polygons comparable component by component, a unified rule is specified for neighboring star selection and vertex ordering: centered on the reference star, within a neighborhood whose angular distance does not exceed $d_{\max}$ ($d_{\max}$ is the maximum search angular distance, with its value given in the parameter table in Section 3.1), the N − 1 stars with the smallest angular distances are selected as neighbors; a right-handed orthonormal basis $\left\{e_{1},e_{2}\right\}$ is established on the tangent plane at the reference star (satisfying $e_{1}\times e_{2}=r_{1}$, with an arbitrary direction for $e_{1}$), and the neighbors are sorted counterclockwise by tangent plane azimuth and numbered sequentially as k=2,⋯,N, with the initial neighbor (k=2) chosen as the selected neighbor with the largest angular distance. This ordering is insensitive to the arbitrary choice of $e_{1}$: a common azimuth offset does not change the cyclic order, and the initial star is uniquely determined when the maximum angular distance is unique. Exactly the same rule is used in catalog preprocessing and online identification to unify the component ordering of the geometric, photometric, and invariant features. When the angular distances of the two longest edges are close and noise may reverse the choice of the initial star, the feature components become misaligned and cause matching to fail for that reference star, and such cases are handled by the fallback mechanism that changes the reference star in Section 2.4.

#### 2.1.1. Geometric Feature Vector g

First, the angular distance between the reference star and a neighboring star is defined (in radians):

(3) $$d_{k}=\arccos(r_{1}^{T}r_{k}),\;k=2,\ldots ,N$$

where $d_{k}$ denotes the angular distance between the reference star and the k-th star; $r_{1}^{T}r_{k}$ denotes the inner product of two unit vectors; and arccos(⋅) denotes the angle operation.

When the focal length and optical distortion have been calibrated, the angular distance $d_{k}$ remains invariant under attitude rotation and retains absolute angular-scale information, so no scale normalization is applied. Normalization by the longest edge would also produce a constant component and render the covariance matrix singular. Differences in physical dimensions and scales among features are handled uniformly by the Mahalanobis distance in Section 2.3.

Consider the angle between adjacent edges. For k=2,…,N, define the unit projection vector of a neighboring star direction on the tangent plane at the reference star:

(4) $$u_{k}=\frac{r_{k}-(r_{1}^{T}r_{k})r_{1}}{\left\|r_{k}-(r_{1}^{T}r_{k})r_{1}\right\|}$$

where $u_{k}$. denotes the normalized projection of $r_{k}$ onto the tangent plane at the reference star and satisfies $\|u_{k}\|=1$.

The angle between two adjacent edges (e.g., k and k+1) in the tangent plane can be written as

(5) $$\alpha_{k}=\arccos(u_{k}^{T}u_{k+1}),\;k=2,\ldots ,N-1$$

where $\alpha_{k}$ denotes the angle formed by two edges with the reference star as the vertex; $u_{k}^{T}u_{k+1}$ denotes the inner product of two unit vectors in the tangent plane.

Stacking the angular distances and included angles in sequence gives the geometric feature vector:

(6) $$g={\left[d_{2},\ldots ,d_{N},\alpha_{2},\ldots ,\alpha_{N-1}\right]}^{T}\in {\mathbb{R}}^{d_{g}}$$

here:

(7) $$d_{g}=(N-1)+(N-2)=2N-3$$

where g denotes the geometric feature vector, which encodes the polygon edge lengths (angular distances) and angles between adjacent edges; $d_{g}$ denotes the geometric feature dimension, consisting of N − 1 angular distances and N − 2 included angles.

#### 2.1.2. Photometric Feature Vector m

To remove the common magnitude zero-point offset caused by overall gain, exposure time, and similar factors, the magnitude difference is defined relative to the reference star:

(8) $$\Delta m_{k}=m_{k}-m_{1},\;k=2,\ldots ,N$$

where $\Delta m_{k}$ denotes the magnitude difference of the k-th star relative to the reference star; using magnitude differences instead of absolute magnitudes helps eliminate the effects of common factors such as overall gain and exposure time on photometric consistency.

The photometric feature vector is constructed as:

(9) $$m={\left[\Delta m_{2},\ldots ,\Delta m_{N}\right]}^{T}\in {\mathbb{R}}^{d_{m}},\;d_{m}=N-1$$

where

m denotes the photometric feature vector, which describes the relative brightness relationships between polygon vertices;

$d_{m}$ denotes the photometric feature dimension, which equals the number of stars other than the reference star, (N−1).

#### 2.1.3. Multi-Order Similarity Invariant Feature Vector Φ

To support rapid k-d-tree indexing in an invariant space in Section 2.4, this section constructs multi-order complex-valued similarity invariants that are insensitive to in-plane rotation, scaling, and translation. Absolute angular-scale information is retained by the geometric features g, whereas Φ characterizes scale-independent shape harmonics, making the two feature types complementary. In the right-handed orthonormal tangent plane basis $\left\{e_{1},e_{2}\right\}$ defined in Section 2.1, each star is mapped to a complex coordinate through gnomonic projection:

(10) $$z_{k}=\;\left(e_{1}^{T}r_{k}\;+\;i\cdot e_{2}^{T}r_{k}\right)\;/\;\left(r_{1}^{T}r_{k}\right),\;k=1,\;\ldots ,\;N$$

where $z_{k}$ denotes the projected coordinate of the k-th star in the complex tangent plane, with the reference star mapped to the origin ($z_{1}$ = 0); i is the imaginary unit; and the denominator $r_{1}^{T}r_{k}$ is the normalization factor of the gnomonic projection.

An ordered star group sequence is formed following the vertex ordering rule in Section 2.1 (the reference star first, followed by neighboring stars in a counterclockwise azimuth order), and the sequence centroid is first removed to eliminate the translation component:

(11) $$w_{k}=z_{k}-\frac{\sum_{j=1}^{N}z_{j}}{N},\;k=1,\ldots ,N$$

The discrete Fourier coefficients of the centered ordered star group sequence are calculated as:

(12) $$c_{j}=\sum_{k=1}^{N}w_{k}\exp\left(-\frac{2\pi ij(k-1)}{N}\right),\;j=0,\ldots ,N-1$$

where $w_{k}$ is the centered complex coordinate, ensuring $c_{0}=0$; $c_{j}$ denotes the j-th-order Fourier coefficient of the star group sequence.

Because the choice of the tangent plane basis $e_{1}$ is arbitrary, the observed and catalog coordinates differ by an in-plane rotation, and calibration residuals may also introduce a small common scale factor. Let this similarity transformation map $z_{k}$ to $s\cdot e^{i\theta }z_{k}+t$ (*s* > 0 is the scale factor, θ is the rotation angle, and t is the translation); then, by the linearity of Equations (11) and (12), all coefficients are multiplied by a common complex factor, transforming $c_{j}$ into $s\cdot e^{i\theta }\cdot c_{j}$. Accordingly, the ratios of the coefficients of different orders to the first-order coefficient are taken to obtain multi-order complex-valued similarity invariants:

(13) $$\varphi_{j}=\frac{c_{j+1}}{c_{1}},\;j=1,\ldots ,J,\;J\leq N-2$$

where $\varphi_{j}$ is the j-th complex-valued similarity invariant and J is the number of retained complex invariants. The common factor $se^{i\theta }$ cancels in the ratio, making the ratio invariant to translation, rotation, and scaling. This paper uses J=N−2, namely J=3 for five stars and J=2 for four stars. When the vertex order is consistent and $c_{1}\neq 0$, these N−2 complex ratios retain the complete similarity-shape information, corresponding to 2N−4 real degrees of freedom; reducing the number of ratios generally loses information. When the magnitude of the first-order coefficient relative to the overall magnitude of all nonzero-order coefficients is less than 10^−8^, the star group is skipped and another group is attempted to avoid numerical instability caused by an excessively small divisor.

Stacking the real and imaginary parts of the invariants of different orders in sequence, the real-valued invariant vector is denoted as:

(14) $$\Phi ={\left[ℜ\varphi_{1},ℑ\varphi_{1},\ldots ,ℜ\varphi_{J},ℑ\varphi_{J}\right]}^{T}\in {\mathbb{R}}^{d_{\Phi }},\;d_{\Phi }=2J$$

where ℜ and ℑ denote the real-part and imaginary-part operations, respectively; Φ is the real-valued invariant vector, with dimension $d_{\Phi }=2J$, corresponding to six and four dimensions for five-star and four-star polygons, respectively.

#### 2.1.4. Joint Feature Vector f

The geometric, photometric, and invariant features are combined to form the joint feature vector:

(15) $$f=\left[\begin{array}{c} g\\ m\\ \Phi \end{array}\right]\in {\mathbb{R}}^{d_{f}},\;d_{f}=d_{g}+d_{m}+d_{\Phi }$$

where

f denotes the joint multi-feature vector, which is the final descriptor used for matching decisions in this paper; $d_{f}$ denotes the total joint feature dimension.

The three subvectors represent the polygon’s geometric shape, brightness relationships, and similarity invariant information, respectively. During catalog preprocessing, the corresponding catalog feature vector $f_{\mathrm{cat}}$ is calculated for each polygon; during online image identification, the observed feature vector $f_{\mathrm{obs}}$ is calculated for each observed polygon. According to the above definitions, the joint feature dimension is $d_{f}$ = 7 + 4 + 6 = 17 for a five-star polygon and $d_{f}$ = 5 + 3 + 4 = 12 for a four-star polygon. This represents a substantial increase over the three-dimensional features of the conventional triangle algorithm and improves the stability of star matching, and although it increases computational complexity, it prevents more severe mismatches.

### 2.2. Statistical Feature Error Model and Covariance Structure

Under a correct match, the observed features can be expressed as:

(16) $$f_{\mathrm{obs}}=f_{\mathrm{cat}}+\delta f$$

where $f_{\mathrm{cat}}$ denotes the true feature vector of the polygon in the catalog (calculated from ideal coordinates and ideal stellar magnitudes); δf denotes the feature error vector, which jointly reflects the effects of perturbation sources such as centroid extraction errors, photometric measurement errors, and residual optical distortion.

It is decomposed here into three parts:

(17) $$\delta f=\left[\begin{array}{c} \delta g\\ \delta m\\ \delta \Phi \end{array}\right]$$

where δg denotes the geometric feature error; δm denotes the photometric feature error; and δΦ denotes the invariant feature error.

Assuming that the errors of the three feature types approximately follow zero-mean Gaussian distributions under a correct match, we have

(18) $$\delta g\sim N(0,\Sigma_{g}),\;\delta m\sim N(0,\Sigma_{m}),\;\delta \Phi \sim N(0,\Sigma_{\Phi })$$

To reduce estimation complexity, this paper does not currently model correlations between the three feature types and approximates the joint covariance in block-diagonal form:

(19) $$\mathrm{Cov}(\delta f)\approx \Sigma_{f}=\left[\begin{array}{ccc} \Sigma_{g} & 0 & 0\\ 0 & \Sigma_{m} & 0\\ 0 & 0 & \Sigma_{\Phi } \end{array}\right]$$

where

$\Sigma_{g}$ denotes the geometric feature covariance, reflecting the statistical dispersion of angular distances and angles between adjacent edges as centroid errors vary;

$\Sigma_{m}$ denotes the photometric feature covariance, estimated from the calibration residuals of relative stellar magnitude differences while retaining correlations caused by the shared reference star;

$\Sigma_{\Phi }$ denotes the invariant covariance;

$\Sigma_{f}$ denotes the block-diagonal approximation of the joint covariance. The covariances of the three feature types are obtained by propagating star measurement uncertainties. Under the specified imaging and noise models, the measurement uncertainties of star coordinates and luminous flux are estimated from the local background and star-spot fitting results in the original star images. Coordinate measurement errors are propagated to angular distances, angles between adjacent edges, and multi-order similarity invariants to obtain the geometric and invariant feature covariances; luminous flux measurement errors are propagated to relative stellar magnitude differences to obtain the photometric feature covariance while retaining correlations introduced by the shared reference star.

### 2.3. Joint Likelihood and Mahalanobis Distance Matching Criterion

Based on the above model, given the catalog polygon features $f_{\mathrm{cat}}$, the deviation of the observed features $f_{\mathrm{obs}}$ from them is:

(20) $$\Delta f=f_{\mathrm{obs}}-f_{\mathrm{cat}}=\left[\begin{array}{c} \Delta g\\ \Delta m\\ \Delta \Phi \end{array}\right]$$

where Δg, Δm, and ΔΦ are the residuals of the geometric, photometric, and real-valued invariant features, respectively. All components are calculated using the same vertex selection and ordering rules to ensure component-wise correspondence between the observed and catalog features. In Equation (8), $\Delta m_{k}$ is the magnitude difference of an individual star relative to the reference star, whereas Δm here is the difference between the observed and catalog relative magnitude vectors.

Conditional on the candidate polygon being a correct match, the joint feature residual is approximately modeled as a zero-mean multivariate Gaussian random vector:

(21) $$\Delta f\sim N(0,\Sigma_{f})$$

where Δf denotes the joint feature residual (observed value minus catalog value); $N(0,\Sigma_{f})$ denotes a multivariate Gaussian distribution with zero mean and covariance $\Sigma_{f}$.

This paper adopts the block-diagonal approximation described in Section 2.2, retaining correlations within the geometric, photometric, and invariant feature blocks separately. Each covariance block is obtained by estimating measurement uncertainties from the local background and star-spot fitting in the original star images and then propagating the errors.

Given a candidate catalog polygon c, the joint feature error is modeled as a zero-mean multivariate Gaussian random vector with the following probability density [13]:

(22) $$p(\Delta f|c)=\frac{1}{{\left(2\pi \right)}^{\nu /2}|\Sigma {|}^{1/2}}\exp\left(-\frac{1}{2}\Delta f^{T}\Sigma^{-1}\Delta f\right)$$

where p(Δf∣C) denotes the probability density of the observed residual conditional on the candidate polygon being the true match; |Σ| denotes the determinant of the covariance matrix; and *v* denotes the joint feature dimension.

The quadratic form in the exponential term is the squared joint Mahalanobis distance. To simultaneously eliminate scale differences among features and account for their covariance relationships, the generalized Mahalanobis distance [14] is adopted, and the joint feature distance is defined as:

(23) $$D^{2}=\Delta f^{T}\Sigma^{-1}\Delta f$$

where

$D^{2}$ denotes the squared Mahalanobis distance of the joint features and is the primary decision statistic used for matching in this paper;

D denotes the normalized length of the observed residual in the joint feature space.

Using the block-diagonal structure (19), the above expression can be decomposed as:

(24) $$\begin{array}{cc} D^{2} & =\left[\begin{array}{ccc} \Delta g^{T} & \Delta m^{T} & \Delta \Phi^{T} \end{array}\right]\left[\begin{array}{ccc} \Sigma_{g}^{-1} & 0 & 0\\ 0 & \Sigma_{m}^{-1} & 0\\ 0 & 0 & \Sigma_{\Phi }^{-1} \end{array}\right]\left[\begin{array}{c} \Delta g\\ \Delta m\\ \Delta \Phi \end{array}\right]\\ & =\Delta g^{T}\Sigma_{g}^{-1}\Delta g+\Delta m^{T}\Sigma_{m}^{-1}\Delta m+\Delta \Phi^{T}\Sigma_{\Phi }^{-1}\Delta \Phi \end{array}$$

Here, the first line is the pure matrix-product form, and the second line is the expanded form, which can be viewed as the sum of three Mahalanobis-distance components:

$D_{g}^{2}=\Delta g^{T}\Sigma_{g}^{-1}\Delta g$ denotes the geometric matching cost;

$D_{m}^{2}=\Delta m^{T}\Sigma_{m}^{-1}\Delta m$ denotes the photometric matching cost;

$D_{\Phi }^{2}=\Delta \Phi^{T}\Sigma_{\Phi }^{-1}\Delta \Phi$ denotes the invariant matching cost.

Accordingly, the sum of the three squared Mahalanobis distances is used as the joint matching cost:

(25) $$D^{2}=D_{g}^{2}+D_{m}^{2}+D_{\Phi }^{2}$$

Here, $D^{2}$ is used for actual matching ranking and decisions. Scale differences among the three feature types and correlations within each block are statistically normalized using their respective inverse covariance matrices.

Each covariance block is obtained by estimating measurement uncertainties from the local background and star-spot fitting in the original star images and then propagating the errors. For candidates of the same order using the same covariance model, minimizing $D^{2}$ is equivalent to maximizing the conditional likelihood given by Equation (22). Let the joint feature dimension be:

(26) $$\nu =d_{g}+d_{m}+d_{\Phi }$$

Then, under the ideal conditions of a correct match, validity of the Gaussian error model above, and a known positive-definite covariance matrix:

(27) $$D^{2}\sim \chi_{\nu }^{2}$$

where $\chi_{\nu }^{2}$ denotes a chi-square distribution with ν degrees of freedom. Denoting the decision threshold for accepting or rejecting a candidate by τ, after specifying the decision parameter α, the threshold can be set to $\tau =F_{\chi_{\nu }^{2}}^{-1}(1-\alpha )$. Under the ideal model above, the probability of rejecting a correct match at this threshold is α. The actual covariance is estimated from a finite sample, and the block-diagonal structure is also an approximation, so the chi-square distribution provides an approximate statistical basis for threshold selection; the parameter α does not represent the algorithm’s actual misidentification rate.

Based on the above, the following matching and decision strategy is adopted in the implementation:

(1) For each observed polygon, coarse screening is first performed using a k-d tree in an invariant space to obtain a set of candidate catalog polygons.

(2) For each candidate, the geometric residual Δg, photometric residual Δm, and invariant residual ΔΦ are calculated, and the joint Mahalanobis distance is computed according to (25).

(3) Only candidates with $D^{2}$ below the threshold τ are retained and sorted in ascending order of distance, and the identification result is determined in conjunction with subsequent consistency verification of star correspondences.

### 2.4. Hierarchical Candidate Retrieval Based on Polygon Order

Based on the joint feature construction and Mahalanobis-distance decisions above, this paper proposes a hierarchical polygon retrieval method that prioritizes five-star polygons and supplements them with four-star polygons. Let the observed star r_1_ be the reference star, with $r_{2}$, $r_{3}$, $r_{4}$, and $r_{5}$ obtained sequentially according to the unified neighboring star selection and ordering rules (see Section 2.1); then the five-star and four-star polygons are represented, respectively, as

(28) $$\left\{\begin{array}{l} P_{5}=\{r_{1},r_{2},r_{3},r_{4},r_{5}\}\\ P_{4}=\frac{r_{v}}{P_{5}}\\ r_{v}=\underset{k\in \{2,\cdots ,5\}}{\arg\;\max\;d_{k}} \end{array}\right.$$

Here, the three neighboring stars in $P_{4}$ are reordered and renumbered according to the rules in Section 2.1. The reference star and its neighbors are all represented by unit line-of-sight vectors in the star sensor coordinate system.

Here, $P_{4}$ is not a simple truncation of $P_{5}$: the four-star polygon consists of the reference star and its three neighbors with the smallest angular distances (i.e., removing from the neighbor set of $P_{5}$ the star with the largest angular distance, $r_{v}$) and is reordered and renumbered according to the rules in Section 2.1; the removed neighbor $r_{v}$ is the “fifth star” used for additional consistency verification at the end of this section.

For N∈{4,5}, the geometric features, photometric features, and multi-order invariants of the polygon are calculated separately and assembled into the joint feature vector:

(29) $$f_{N}={\left[\begin{array}{ccc} g_{N}^{T} & m_{N}^{T} & \Phi_{N}^{T} \end{array}\right]}^{T}$$

Here, $g_{N}$ describes the angular-distance and included-angle relationships between the reference star and its neighbors, $m_{N}$ describes the magnitude difference of each neighbor relative to the reference star, and $\Phi_{N}$ represents the multi-order invariant features. Four-star and five-star polygons use the same feature organization, differing only in the number of vertices and feature dimensions, thereby ensuring consistent physical interpretations and decision criteria at both retrieval levels.

During catalog preprocessing, five-star and four-star polygon feature retrieval trees $T_{5}$ and $T_{4}$ are constructed separately, and the corresponding star identifier combinations and joint features are stored. During online identification, the invariant features of the observed polygon are first used for nearest-neighbor search in the corresponding k-d tree to obtain the initial candidate set $C_{N}^{I}$:

(30) $$C_{N}^{I}=\mathrm{KNN}\left(\Phi_{N}^{\mathrm{obs}},T_{N}\right),\;N\in \{4,5\}$$

The Mahalanobis distance between the observed polygon and each candidate catalog polygon is then calculated, and the candidate set $C_{N}^{S}$ satisfying the threshold condition is retained:

(31) $$C_{N}^{S}=\left\{c\in C_{N}^{I}:D_{w,N}^{2}(c)\leq \tau_{N}^{2}\right\}$$

where c denotes a candidate catalog polygon, $D_{w,N}^{2}(c)$ is the joint feature distance defined in Equation (25), and $\tau_{N}^{2}$ is the screening threshold for the corresponding order. This process uses a hierarchical procedure combining “coarse screening with a k-d tree in invariant space + fine discrimination using joint features” to avoid comparing all catalog polygons individually.

The algorithm first performs five-star polygon retrieval. When a unique reliable candidate is obtained in $C_{5}^{S}$ and passes subsequent consistency verification of star correspondences, the identification result is output. Because the five-star features simultaneously incorporate geometric, photometric, and invariant constraints from five observed stars, they can improve candidate polygon separability during retrieval and reduce the number of candidates entering full-field verification.

When five-star retrieval produces no candidate, multiple candidates, or a candidate that fails consistency verification, the algorithm proceeds to four-star polygon retrieval. To compensate for the reduced constraints caused by the lower polygon order, the fifth star, which is not used to construct the four-star features, provides additional verification of four-star candidates. Let the directions of the four catalog stars corresponding to candidate c be $s_{i}^{\left(c\right)}$, i=1,…,4, and let the set of catalog stars in the candidate neighborhood without established correspondences be $S_{c}$; then the fifth-star angular-distance consistency error is defined as $E_{5}(c)$:

(32) $$E_{5}(c)=\min_{s\in S_{c}}\frac{1}{4}\sum_{i=1}^{4}\left|\arccos(r_{v}^{T}r_{i})-\arccos(s^{T}s_{i}^{\left(c\right)})\right|$$

Accordingly, the four-star candidate set after additional verification is expressed as $C_{4}^{V}$:

(33) $$C_{4}^{V}=\left\{c\in C_{4}^{S}:E_{5}(c)\leq \tau_{v}\right\}$$

Here, $\tau_{v}$ is the fifth-star consistency verification threshold. When a unique reliable candidate is obtained in $C_{4}^{V}$ and passes full-field star verification, star identification is completed; otherwise, the reference star is changed, and the observed polygon is reconstructed. Thus, the five-star path reduces the candidate space using higher-dimensional joint constraints, whereas the four-star path maintains constraint redundancy in candidate decisions through verification with one additional star. This hierarchical structure avoids layer-by-layer expansion of observed star combinations and reduces the number of candidates entering full-field verification while maintaining identification availability. Section 3 compares the identification performance and runtime efficiency of the proposed method using measured star images.

### 2.5. Analysis of Algorithm Characteristics

The polygon-based star identification algorithm proposed in this paper has the following main characteristics:

(1) Extension from a single shape invariant to a fused feature space. Conventional methods mainly rely on a single or a small number of similarity invariants for candidate retrieval and matching decisions, essentially searching a low-dimensional invariant space with limited feature information and susceptibility to candidate ambiguity under noise. This paper jointly embeds the polygon’s geometric shape (edge lengths expressed as angular distances and angles between adjacent edges), relative brightness relationships between vertices, and multi-order similarity invariants into a $d_{f}$-dimensional real vector space ${\mathbb{R}}^{d_{f}}$ to form a joint descriptor, improving the completeness of the description of a polygon while increasing the separability of different polygons in feature space.

(2) Separate statistical covariance models for the feature types, combined in a block-diagonal structure. The sensitivity of geometric, photometric, and invariant features to perturbation sources such as centroid errors and photometric noise is characterized by $\Sigma_{g}$, $\Sigma_{m}$, and $\Sigma_{\Phi }$, respectively, and a block-diagonal structure is adopted for the joint covariance matrix. This modeling approach preserves the statistical properties of the physical quantities and allows the joint matching cost to be decoupled into three physically meaningful and individually interpretable components, providing a basis for feature contribution analysis and parameter tuning.

(3) Integration of different features using the joint Mahalanobis distance. The covariance-normalized matching costs of the three feature types are added to form the joint cost $D^{2}$ for candidate ranking and screening. Under the approximately Gaussian error and block-diagonal covariance models, the decision threshold is set with reference to the chi-square distribution.

(4) Establishment of a hierarchical candidate retrieval architecture based on polygon order. The algorithm prioritizes five-star polygons and uses high-dimensional joint features to substantially reduce the candidate space during retrieval, significantly decreasing the number of candidates entering lengthy full-field verification; when the five-star path cannot produce a reliable candidate, it falls back to the four-star index and applies a fifth-star consistency check to maintain constraint redundancy. This architecture avoids search-space expansion caused by layer-by-layer combination enumeration, maintains a subsecond P95 runtime for successful frames in all eleven data environments, and produces no timeouts in all 4092 frame-wise solutions (372 frames × 11 environments), in contrast to combination-search methods whose runtimes increase substantially under false-star and combined perturbations.

## 3. Comparative Experiments

Using measured star images, this section compares and evaluates the proposed algorithm in terms of identification rate, misidentification, runtime distribution, and search overhead.

### 3.1. Experimental Setup and Evaluation Criteria

The experiment uses 372 star images acquired by a star sensor at a fixed ground station on 28 November 2025 from 19:29:13.680 to 19:38:03.380 (UTC+8). The exposure time was 50 ms, and there was no external ambient-light interference during acquisition. Star centroids were taken directly from the star sensor output without software postprocessing. Each frame underwent independent all-sky lost-in-space identification. Figure 1 shows the data acquisition site and equipment used in this experiment, where (a) presents all equipment and (b) shows equipment details.

The comparison algorithms are configured as follows: the triangle algorithm uses angular distances and included angles formed by an observed star and its two nearest neighbors as features, sets the angular-distance tolerance to the value corresponding to 3σ, and introduces a verification star to resolve ambiguity; the pyramid algorithm uses a k-vector for star-pair retrieval, performs matching with a three-star primitive, and resolves ambiguity through fourth-star verification.

The geometric polygon algorithm uses a two-dimensional invariant baseline based on the Ramos method, forms a five-star configuration from the reference star and its four neighbors, and generates up to 15 candidate configurations by replacing neighboring stars; the nearest neighbor determines the starting point of the ordering, and the remaining neighbors are sorted counterclockwise by tangent plane azimuth. The real and imaginary parts of a single j=1 complex invariant are used as two-dimensional retrieval features for a rectangular range search with preset tolerances in a k-d tree, followed by candidate verification using four-point and three-point internal polygons and field-of-view voting across different observed reference stars. Candidates undergo further affine tangent plane fitting and full-field matching verification with an angular-distance matching tolerance of 0.18°, and acceptance requires at least $\max[5,\min(8,n_{\mathrm{obs}})]$ matched stars and a matching residual RMS no greater than 0.12°, where $n_{\mathrm{obs}}$ is the number of observed stars in the current frame.

The experimental data consist of the 372 measured star images described above; the star catalog and all precomputed indices (the triangle feature index, four-star/five-star polygon indices, star-pair k-vector, and verification catalog containing 20,678 stars) were configured before the experiment.

The decision thresholds of the proposed algorithm are set using the chi-square approximation in Section 2.3, and sensitivity tests examine decision parameter α values of 0.0001, 0.001, and 0.01, nearest-neighbor count k values of 100, 200, and 400, and fifth-star consistency threshold $\tau_{v}$ values of 0.06°, 0.12°, and 0.24°, respectively. Based on the test results, α = 0.001, k = 400, and τ_v = 0.12° are selected, with the complete parameters listed in Table 1.

A unified frame-level evaluation criterion is used for all algorithms: identification is considered correct when the algorithm accepts the result and the error in the boresight direction relative to the reference value does not exceed 0.2°. This criterion evaluates the consistency of the boresight direction and does not assess rotation about the boresight or full three-axis attitude accuracy.

The identification rate is the proportion of correctly identified frames among valid frames, and the number of misidentifications is the number of frames for which the algorithm outputs an incorrect solution. A timeout is recorded when no solution is produced within 60 s for a single frame. Correctly identified frames without a timeout are defined as successful frames, and the median (P50) and 95th percentile (P95) of their solution runtimes are calculated. The identification failure rate is the sum of rejected, misidentified, and timed-out frames divided by the number of valid frames.

### 3.2. Runtime Statistics

Figure 2 presents the empirical cumulative distribution of frame-wise solution runtimes for the four algorithms on 372 star images under unperturbed conditions (with a logarithmic horizontal axis), where curves farther to the left and steeper indicate shorter runtimes and smaller frame-to-frame variations. The proposed algorithm has a P50/P95 of 241.4/274.2 ms, representing reductions of 20.5% and 49.6%, respectively, relative to the triangle algorithm (303.6/543.6 ms), and 86.3% and 97.9%, respectively, relative to the pyramid algorithm (1765.6/12,886.0 ms), with larger reductions at the higher percentile.

Within a 300 ms limit, the proposed algorithm completes the solution for 97.3% of frames, whereas the triangle and pyramid algorithms complete only 49.5% and 3.5%, respectively. The proposed algorithm completes 99.7% of frames within 1 s, with a maximum single-frame runtime of only 1.1 s; the large combination-search overhead of the pyramid algorithm shifts its entire curve to the right and produces a pronounced long tail, with a maximum single-frame runtime of 45.2 s. Overall, the proposed algorithm outperforms the triangle and pyramid algorithms in both runtime efficiency and runtime stability.

### 3.3. Identification Performance in Eleven Data Environments

To examine the algorithm’s adaptability to typical observational degradation, eleven data environments are constructed from the 372 baseline frames: an unperturbed baseline; Gaussian centroid noise ($\sigma_{p}$ = 0.5 and 1.0 px); random star dropout (10% and 20%); false-star injection (10% and 20%, with at least eight true stars in the original frame); photometric perturbation (star positions remain unchanged, and lognormal multiplicative perturbations are applied to luminous flux, with logarithmic standard deviations $\sigma_{F}$ = 0.3, 0.5, and 1.0); and moderate combined perturbations incorporating all four perturbation types. Perturbations for each condition are generated with fixed random seeds and applied directly to centroid coordinates and luminous flux inputs, with exactly the same inputs provided to all four algorithms.

Figure 3 illustrates how the different perturbations act using a measured star image containing 19 observed stars. Under centroid noise $\sigma_{p}$ = 1.0 px, the number of stars remains unchanged, and the mean, RMS, and maximum displacements are 1.136 px, 1.288 px, and 2.338 px, respectively, simulating imaging noise and centroid extraction errors; 20% star dropout randomly removes four true stars to simulate missed detections of faint stars and losses near the field-of-view boundary; 20% false-star injection adds four false stars at random positions, with photometry sampled and scaled from true stars, simulating hot pixels, noise spikes, and stray light; under photometric perturbation $\sigma_{F}$ = 1.0, the median photometric ratio is 1.031, with extremes of 0.207 and 7.560; combined perturbations simultaneously apply lower-amplitude centroid noise (RMS 0.833 px), star dropout, false stars, and photometric perturbation to better approximate practical engineering inputs.

The identification rates and misidentification counts of the four algorithms under each condition are listed in Table 2, and the trade-off between failure rate and runtime is shown in Figure 4.

Table 2 shows that the proposed algorithm achieves the highest identification rate under the unperturbed baseline and the other seven conditions that do not involve photometric perturbation alone. Its unperturbed identification rate is 92.74%, exceeding those of the triangle, pyramid, and geometric polygon algorithms by 26.07, 7.79, and 11.29 percentage points, respectively; it maintains 92.20% under centroid noise and false-star conditions, achieves 88.98% and 86.02% under 10% and 20% star dropout, respectively, and remains at 84.14% under combined perturbations, showing good adaptability to geometric perturbations within the tested range.

The triangle algorithm is sensitive to centroid noise: as $\sigma_{p}$ increases to 0.5 and 1.0 px, its identification rate decreases from 66.67% in the unperturbed case to 31.18% and 14.78%, and misidentifications increase from 12 frames to 27 and 55 frames; under 10% and 20% false stars, its identification rate decreases to 58.60% and 54.03%, and misidentifications increase to 28 and 49 frames; under combined perturbations, its identification rate is only 25.00%, with 35 misidentified frames. The algorithm is computationally fast, with its points mainly on the right side of Figure 4, but its identification rate is only 14.78–67.47% and misidentifications occur under all conditions, indicating relatively limited identification accuracy and perturbation tolerance.

The pyramid algorithm achieves identification rates of 80.11–84.95% across the environments without any misidentifications, benefiting from fourth-star uniqueness verification; however, its successful-frame P95 runtime reaches 4.770–18.417 s, with a median of 11.755 s, higher than that of the triangle algorithm, and one, four, and four frames reach the 60 s timeout under 10% false stars, 20% false stars, and combined perturbations, respectively, indicating that false-star-induced expansion of candidate combinations substantially increases its search overhead.

Under photometric perturbation, because the three comparison methods use photometry-independent geometric features, the identification rates and misidentification counts of the triangle, pyramid, and geometric polygon algorithms remain the same as in the unperturbed case (66.67%, 84.95%, and 81.45%, respectively), whereas the proposed algorithm’s identification rate decreases to 82.80%, 81.18%, and 77.96% as $\sigma_{F}$ increases (without misidentifications), indicating greater sensitivity to photometric perturbation and an identification rate advantage that does not extend to all test conditions. In addition, the proposed algorithm produces one, three, and two misidentified frames under 10% star dropout, 20% false stars, and combined perturbations, respectively, so erroneous-match control still requires improvement.

Figure 4 further presents 95% interval estimates of the identification rates and failure type breakdowns for the four algorithms in each environment. Each algorithm completes 4092 frame-wise evaluations, with the Fusion feature polygon algorithm producing 3581 correct identifications (87.51%), six misidentifications (0.15%), 505 rejections, and no timeouts; the pyramid algorithm produces 3437 correct identifications (83.99%), no misidentifications, 646 rejections, and nine timeouts; the geometric polygon algorithm produces 3028 correct identifications (74.00%), 1064 rejections, and no misidentifications or timeouts; the triangle algorithm produces 2159 correct identifications (52.76%), 257 misidentifications (6.28%), 1676 rejections, and no timeouts. All categories are counted according to the criteria defined in this paper.

Neither the pyramid nor the geometric polygon algorithm produces misidentifications, but the latter shows a marked increase in rejections under star dropout, with only 150 correct frames and 222 rejected frames at 20% dropout. The triangle algorithm has the highest misidentification proportion and degrades substantially under centroid noise and combined perturbations, with only 55 correct frames, 55 misidentified frames, and 262 rejected frames out of 372 under 1.0 px centroid noise. The Fusion feature polygon algorithm has the highest overall proportion of correct identifications and the lowest rejection proportion in the illustrated configuration, with rejection remaining its main failure type; under combined perturbations, it produces 313 correct frames, two misidentified frames, and 57 rejected frames.

Under photometric perturbations $\sigma_{F}$ = 0.30, 0.50, and 1.00, the full joint-scoring configuration produces 308, 302, and 290 correct frames, identification rates of 82.80%, 81.18%, and 77.96%, and 64, 70, and 82 rejections, respectively, without any misidentifications or timeouts. Photometric perturbations change the relative brightness relationships between observed stars and catalog stars, reducing the reliability of photometric features for distinguishing candidates; changes in bright-star ranking also affect the selection of the reference star and the star groups used for matching, further influencing candidate construction and retrieval. Covariance normalization can adjust the contribution of photometric residuals to the total score but cannot recover the perturbed photometric information. In the current configuration, these effects manifest as increased rejections and reduced identification rates, reflecting the photometric sensitivity associated with using relative brightness to assist matching.

Figure 5 presents the trade-off distribution between identification failure rate (horizontal axis) and successful-frame P95 runtime (vertical axis, logarithmic scale) for the four algorithms in eleven data environments, with both quantities defined in Section 3.1 and point labels 1–11 corresponding to the row order in Table 2; points farther toward the lower left indicate lower failure rates and shorter runtimes.

The proposed algorithm’s points cluster in the region of low failure rate and short runtime: across the eleven environments, its failure rate is 7.26–22.04% (median 11.02%), and its successful-frame P95 runtime is 0.245–0.811 s (median 0.426 s); its failure rate is the lowest among the four algorithms in eight environments, and its P95 runtime is lower than those of the pyramid and geometric polygon algorithms in all environments and lower than that of the triangle algorithm in seven environments. The triangle algorithm has a short runtime (0.386–0.537 s) but a failure rate of 32.53–85.22%, with its points on the right side of the figure, indicating high speed but insufficient robustness; the pyramid algorithm has a failure rate of 15.05–19.89% but a P95 runtime of 4.770–18.417 s, with its points in the upper part of the figure; the geometric polygon algorithm has a failure rate of 18.55–59.68% (median 18.82%) and a P95 runtime of 1.206–8.012 s (median 1.581 s), with the failure rate rising to 59.68% under 20% star dropout and the P95 runtime reaching 8.012 s and 7.152 s under 20% false stars and combined perturbations, respectively, indicating that missing stars weaken its identification capability while false stars and combined perturbations increase its high-percentile runtime.

In the unperturbed environment, the failure rates of the proposed, triangle, pyramid, and geometric polygon algorithms are 7.26%, 33.33%, 15.05%, and 18.55%, respectively, and their P95 runtimes are 0.273, 0.467, 5.071, and 1.520 s, respectively; relative to the latter three algorithms, the proposed algorithm reduces failure rates by 26.07, 7.79, and 11.29 percentage points and P95 runtimes by 41.5%, 94.6%, and 82.1%, respectively. Under moderate combined perturbations, the four algorithms have failure rates of 15.86%, 75.00%, 18.01%, and 38.17%, respectively, and P95 runtimes of 0.426, 0.508, 18.417, and 7.152 s, respectively.

Under 20% false stars, the proposed algorithm’s P95 runtime increases to 0.715 s, slightly above the triangle algorithm’s 0.537 s, but its failure rate is only 7.80%, below the triangle algorithm’s 45.97% and the geometric polygon algorithm’s 19.35%. Under the three photometric perturbation conditions, the proposed algorithm’s failure rates are 17.20%, 18.82%, and 22.04%, respectively, all higher than those of the pyramid algorithm and, for the latter two levels, also higher than those of the geometric polygon algorithm, so its failure rate advantage does not extend to photometric perturbation conditions. The pyramid algorithm’s timed-out frames are included in the failure rate but excluded from the successful-frame P95, so its high runtime is not caused by timeout truncation. Overall, the proposed algorithm provides the best balance between speed and robustness (with points farthest toward the lower left) and the greatest potential for real-time applications.

### 3.4. Solution Runtime Distribution and Search Overhead Analysis

Figure 6 presents the distributions of successful-frame solution runtimes in each environment. The proposed algorithm has a median runtime of 0.140–0.299 s and a P95 of 0.245–0.715 s: the P95 remains at 0.268–0.281 s under unperturbed, centroid noise, and photometric perturbation conditions and increases to 0.439, 0.715, and 0.426 s under 10% false stars, 20% false stars, and combined perturbations, respectively, indicating that false stars produce only a limited long tail by increasing candidate verification. The triangle algorithm has a P95 of 0.386–0.537 s, but its number of successful frames decreases sharply under centroid noise (Figure 4), so stable runtimes do not imply robustness. The pyramid algorithm has a median runtime of 1.436–4.173 s and a P95 of 4.770–18.417 s, with both the boxes and P95 shifting markedly upward under star dropout, false stars, and combined perturbations. The geometric polygon algorithm has a median runtime of 0.905–6.277 s and a P95 of 1.206–8.012 s: its P95 under photometric perturbation is similar to that in the unperturbed case (1.520–1.581 s), increases to 1.896–3.545 s under centroid noise, and reaches 7.150–8.012 s under false stars and combined perturbations; although its median and P95 are lowest under 20% star dropout (0.905 s and 1.206 s), its identification rate simultaneously decreases from 81.45% to 40.32%, with the shorter runtime resulting from fewer successful samples. The successful-frame P95 values pooled across environments are 0.389 s for the proposed algorithm, 0.496 s for the triangle algorithm, 12.623 s for the pyramid algorithm, and 6.814 s for the geometric polygon algorithm.

Figure 7 (left) presents the search-path composition of the proposed algorithm: of all 4092 frame-wise solutions, 81.16% are completed through the direct five-star path, 18.50% enter the four-star fallback, and only 0.34% (14 frame-wise solutions, all under 20% star dropout) produce no output because of insufficient available stars (counted as rejections). In the other environments, the direct five-star path accounts for 80.38–82.26%, indicating that ordinary perturbations do not change the main workflow; under 20% star dropout, the direct five-star proportion decreases to 75.27% and the four-star fallback increases to 20.97%, showing more frequent use of the fallback branch when stars are missing, consistent with disruption of five-star combinations by star dropout. Figure 7 (right) shows the median/P95 full-field verification counts for correct frames: the triangle algorithm has 38/120 (close to the preset limit), with runtime strongly correlated with verification count (Spearman ρ = 0.881), and the many verifications caused by candidate ambiguity do not yield a higher identification rate; the pyramid algorithm has 1/8 (ρ = 0.727), with few verifications but high unit costs for candidate generation and pairwise expansion; the geometric polygon algorithm has 1/3 (correlation coefficient 0.525), with 86.96% of correct frames requiring verification of only one candidate and a median runtime of 1.336 s; the proposed algorithm has 1/36 (ρ = 0.593), with most correct frames requiring only one verification and subsecond runtimes despite long tails under false stars and combined perturbations. Thus, hierarchical retrieval reduces the candidate space while maintaining a low unit verification cost, balancing identification rate and speed.

### 3.5. Mechanism Analysis Using Representative Frames

To illustrate the identification mechanism of the proposed method, Figure 8 selects four representative cases: “correcting misidentification, reducing rejection, outperforming the geometric polygon algorithm, and excluding false stars”. The same configuration is used in all cases, and all star-by-star matches of the proposed method are checked against the reference catalog. White points denote true observed stars, red points denote injected false stars, red × symbols mark confirmed star-by-star mismatches of the comparison algorithms, and whole-frame rejections are indicated in the subplot titles.

In case (a), the triangle algorithm still outputs an incorrect solution after full-field verification of 120 candidates, taking 0.594 s, with all eight matches inconsistent with the reference identities; through the direct five-star path, the proposed method correctly matches all 17 true stars after verifying only one candidate, taking 0.240 s and combining mismatch suppression with low verification overhead. In case (b), the pyramid algorithm verifies nine candidates in 13.499 s but obtains only seven matches and rejects the frame because it does not meet the acceptance requirement of eight matches; the Fusion feature polygon algorithm correctly matches eight of the nine observed stars in 0.418 s, demonstrating how the fallback path reduces rejections. In case (c), the geometric polygon algorithm retrieves 12,796 candidates, none of which pass preliminary screening, and rejects the frame after 3.815 s; through four-star fallback, two fifth-star consistency checks, and one full-field verification, the proposed method correctly matches all nine observed stars in 0.534 s, showing that more candidates do not necessarily produce effective identification and that joint feature constraints facilitate correct solutions. Case (d) contains eight true stars and one injected false star, and the geometric polygon algorithm outputs nine matches in 0.513 s, including one false-star mismatch; through four-star fallback, one fifth-star consistency check, and one full-field verification, the proposed method correctly matches the eight true stars and excludes the false star in 0.486 s. Because the geometric polygon algorithm still satisfies the boresight-error criterion for this frame, this case mainly demonstrates improved reliability of star-by-star matching.

## 4. Conclusions

For all-sky lost-in-space identification with star sensors, this paper proposes a polygon-based star identification algorithm that combines geometric, photometric, and multi-order similarity invariant features, using stellar image-plane positions and relative brightness to establish correspondences between observed stars and catalog stars and provide inputs for multi-star attitude determination. The method normalizes different residual types using block-diagonal covariance matrices, screens candidates by the joint Mahalanobis distance, and adopts a hierarchical retrieval procedure with five-star priority, four-star fallback, and fifth-star consistency verification. Based on experiments using 372 measured star images in eleven data environments, the main conclusions are as follows:

(1) Algorithm identification rate. The proposed method achieves identification rates of 77.96–92.74% in eleven environments and outperforms the three comparison algorithms under all eight non-photometric perturbation conditions, including the unperturbed case, demonstrating the effectiveness of its candidate discrimination and multi-star consistency constraints. Under photometric perturbations σF = 0.30, 0.50, and 1.00, its identification rates are 82.80%, 81.18%, and 77.96%, respectively, which are 1.34 percentage points higher, 0.27 percentage points lower, and 3.49 percentage points lower, respectively, than those of the geometric polygon algorithm without photometric features, indicating comparable or better performance under weak and moderate perturbations and some degradation under strong perturbations while still correctly identifying most test frames and maintaining a high proportion of valid outputs.

(2) Algorithm efficiency and stability. Efficiency and failure modes. The proposed method maintains a successful-frame P95 runtime below 1 s under all conditions, with reductions of 41.5%, 94.6%, and 82.1% relative to the triangle, pyramid, and geometric polygon algorithms, respectively, in the unperturbed case; under combined perturbations, the successful-frame P95 runtimes of the improved algorithm, the pyramid algorithm, and the geometric polygon algorithm are 0.426 s, 18.417 s, and 7.152 s, respectively. Across 4092 frame-wise solutions, only six misidentifications (0.15%) occur, with no timeouts, and failures mainly take the form of detectable rejections. In comparison, the pyramid algorithm produces no misidentifications but has many rejections, some timeouts, and runtimes ranging from seconds to tens of seconds; the triangle algorithm has comparable runtimes but produces 257 misidentifications. The proposed method is the only one of the four algorithms to combine a low failure rate with a short runtime.

(3) Applicability and limitations. The proposed method is suitable for single-frame lost-in-space identification when sufficient valid stars are present in the field of view to establish reliable multi-star correspondences; its adaptability to strong photometric perturbations remains limited; the existing experiments mainly cover stationary observations or observations with small-angle rotations, and performance under rapid rotation and strong stray light has not yet been verified.

(4) Future work. Optimize the use of low-reliability photometric information; test identification performance during continuous tracking; conduct system-level simulations of simultaneous identification with dual sensors and dual fields of view to assess observation availability and the effects of misidentification.

## Figures and Tables

**Figure 1:**
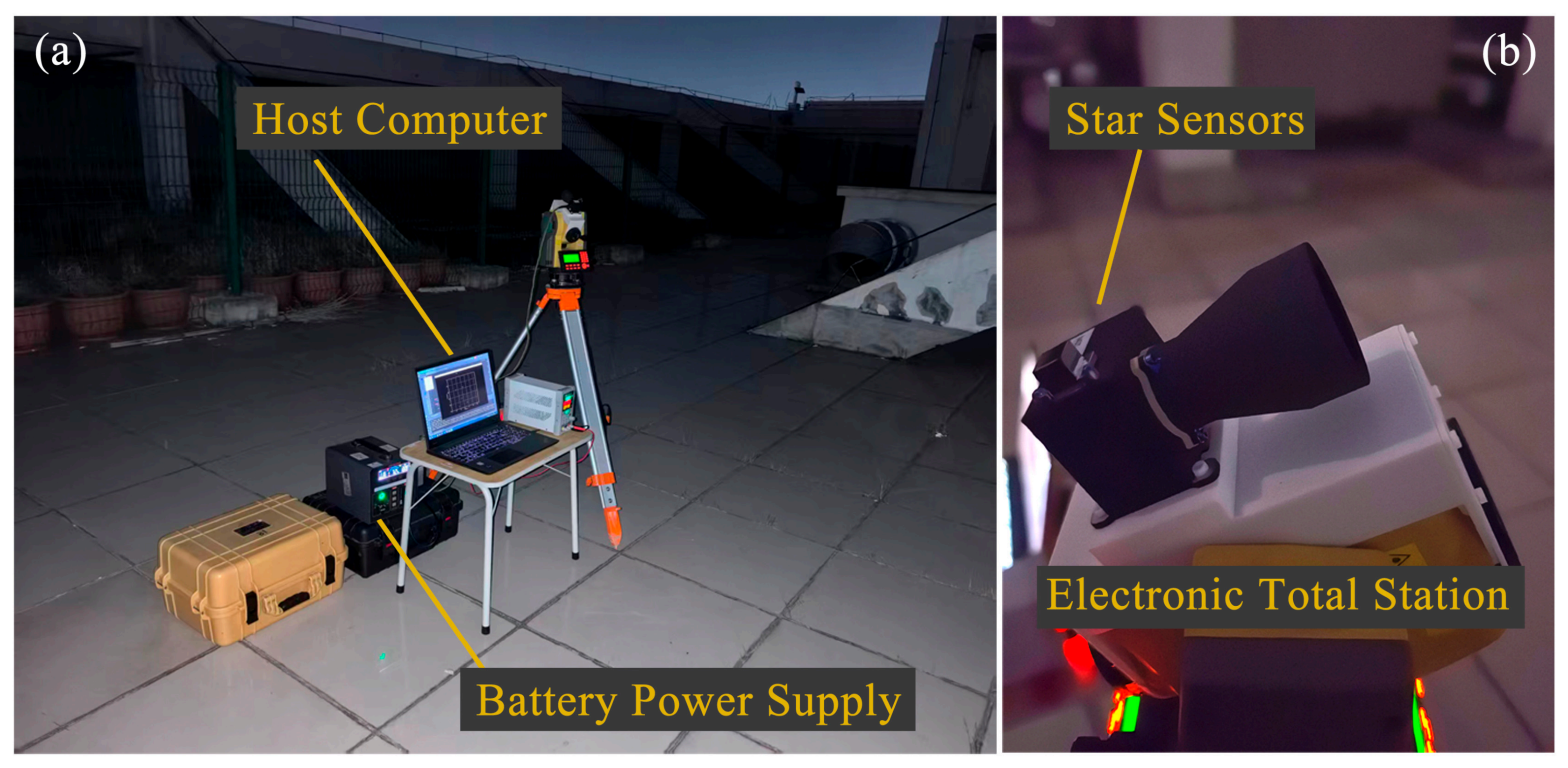
Data acquisition site and equipment. (**a**) Data acquisition site and overall experimental setup (**b**) Close-up of the star sensors mounted on the electronic total station.

**Figure 2:**
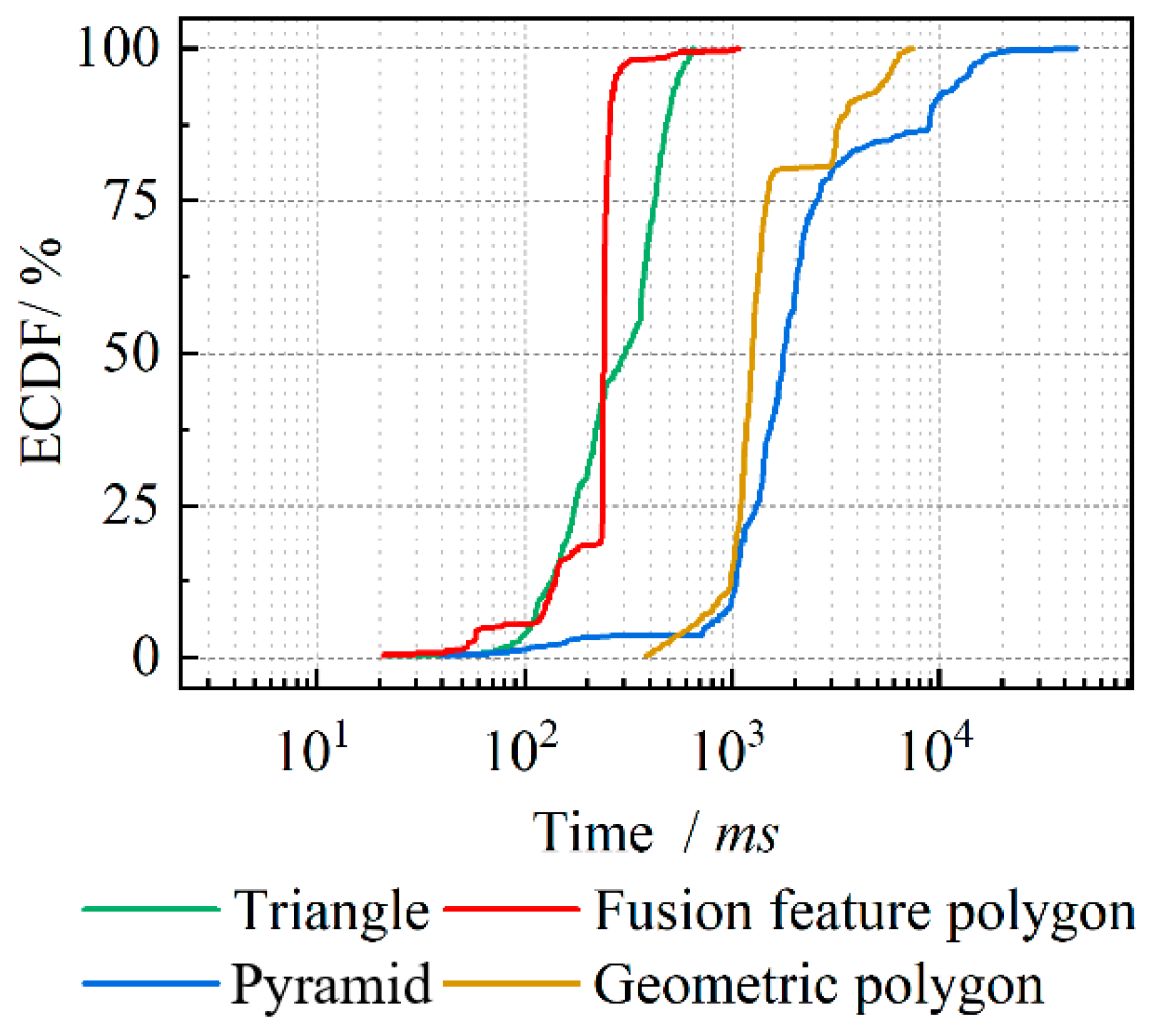
Solution efficiency curves of four star identification algorithms.

**Figure 3:**
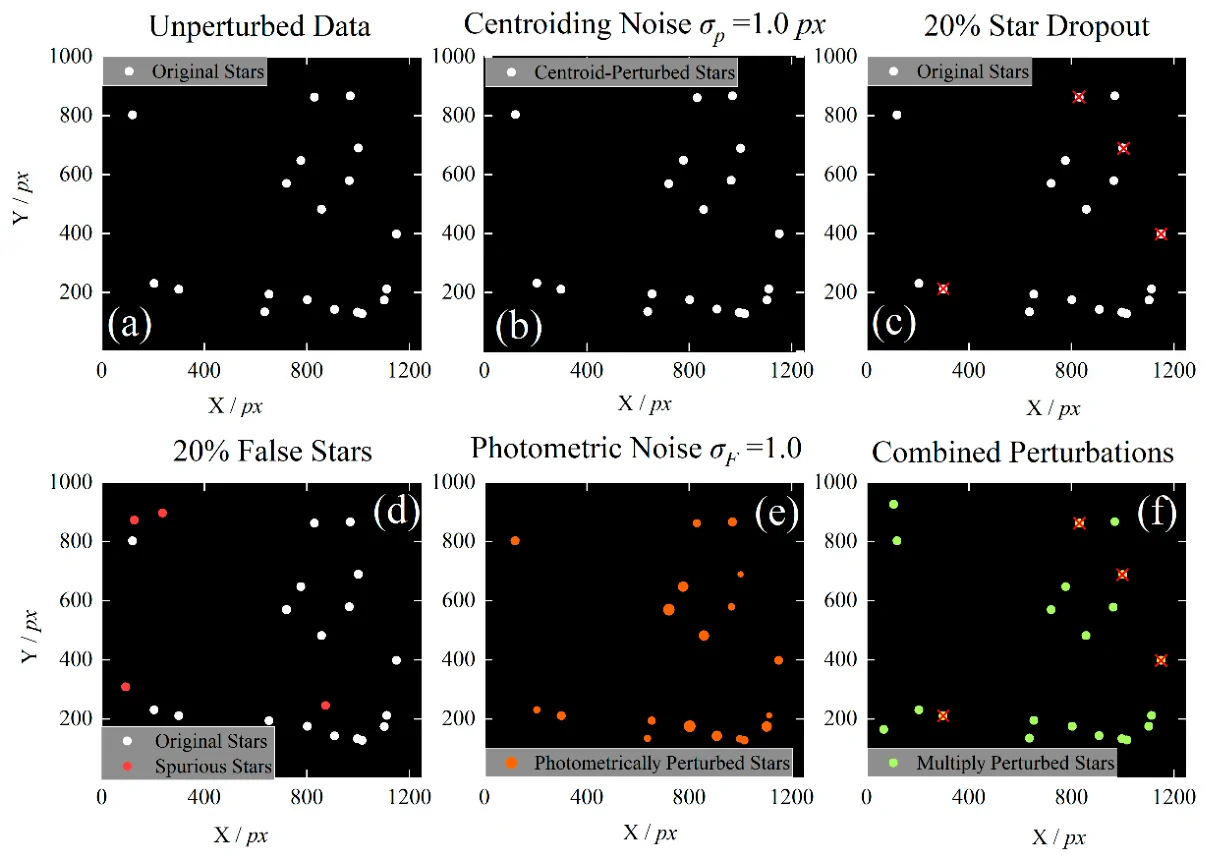
Single-frame examples of six representative perturbation types. (**a**) Unperturbed reference star image. (**b**) Gaussian centroid noise ($\sigma_{p}$ = 1.0 px). (**c**) 20% random star dropout. (**d**) 20% false-star injection. (**e**) Photometric perturbation ($\sigma_{F}$ = 1.0). (**f**) Combined perturbations. The crossed-out circles in panels (**c**,**f**) mark the original positions of stars removed to simulate star dropout.

**Figure 4:**
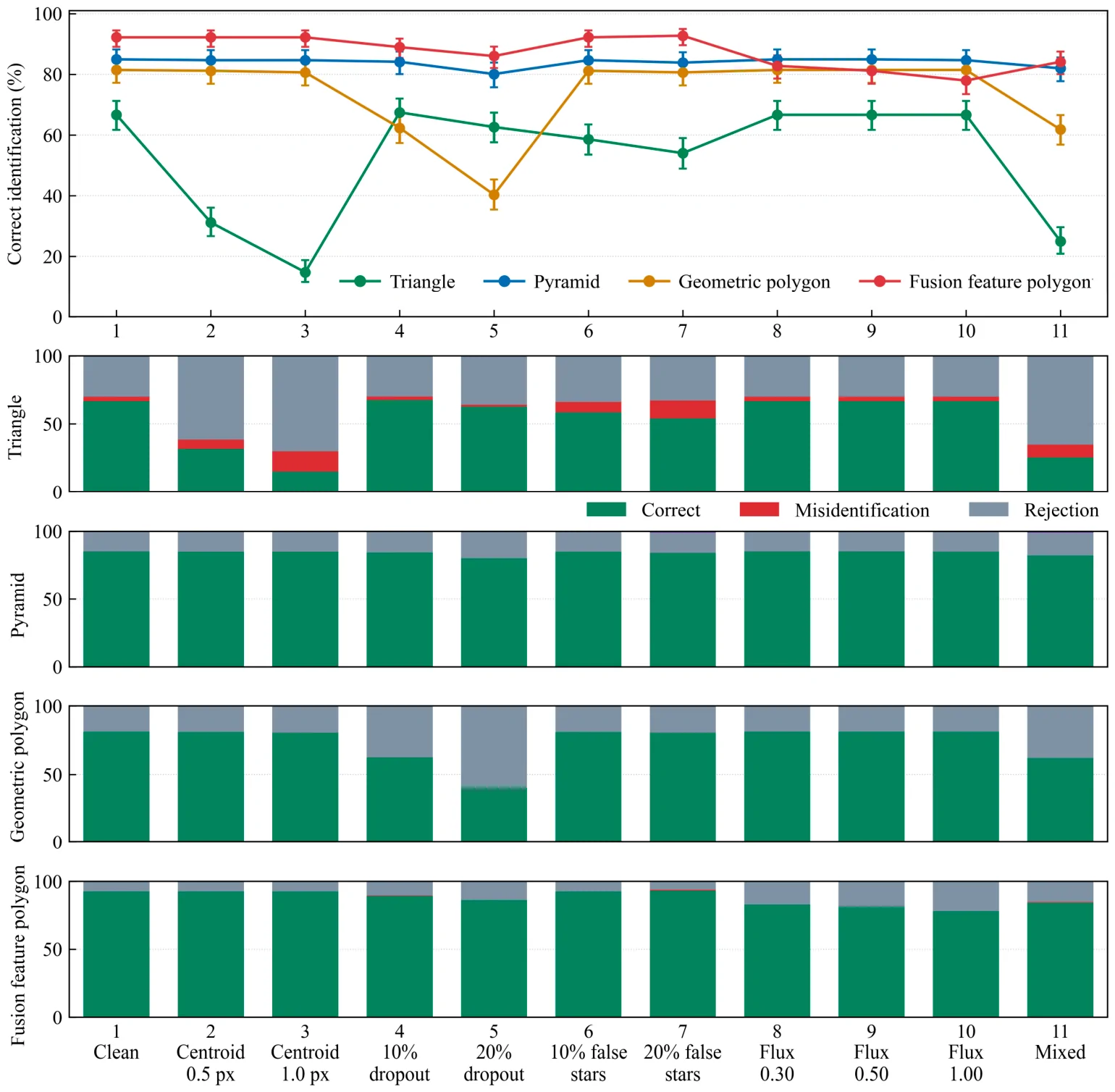
Identification rate responses and failure type breakdowns of four algorithms in eleven data environments.

**Figure 5:**
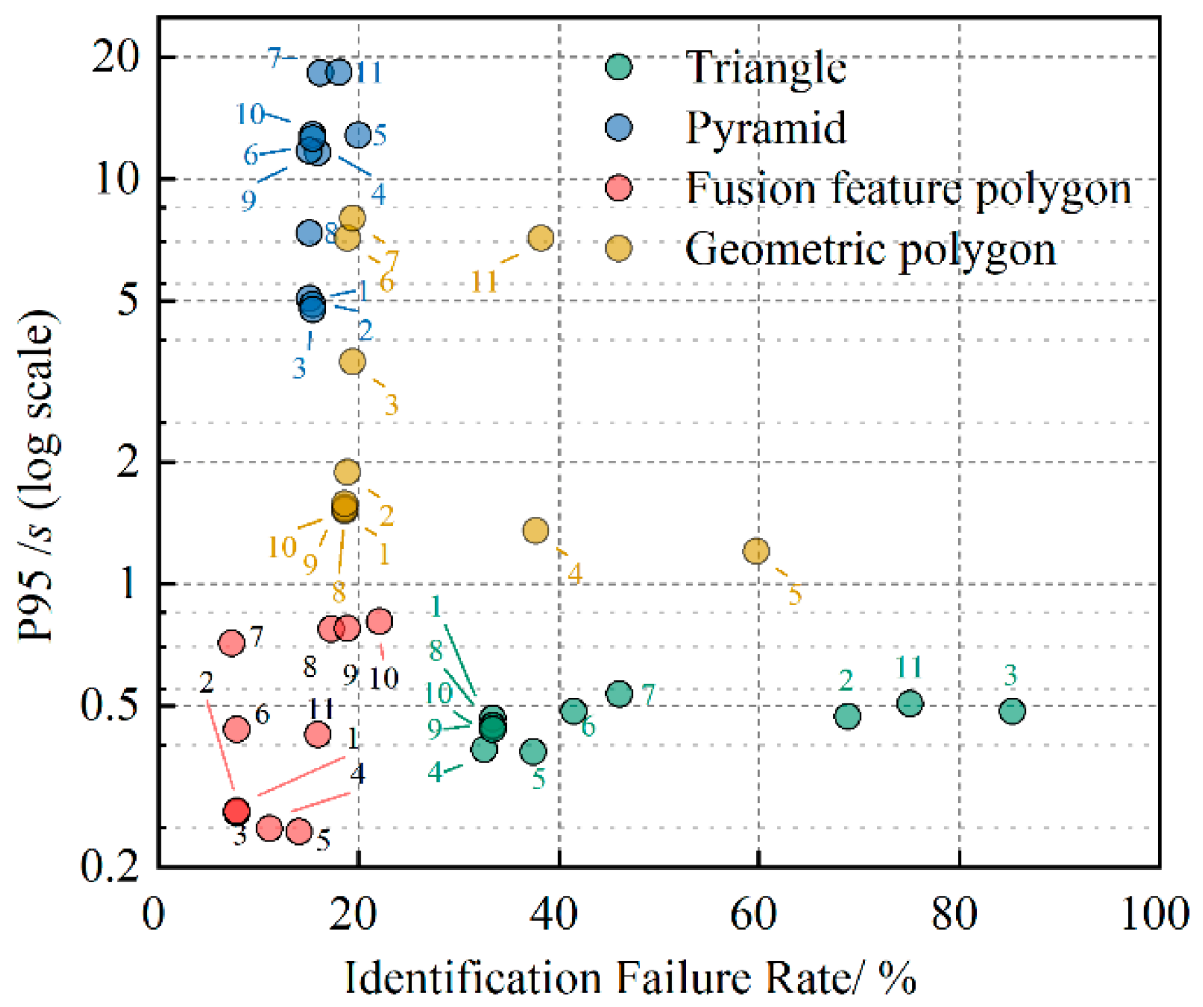
Solution efficiency statistics of four algorithms in different data environments. The number next to the circle here represents the star map recognition performance of this algorithm under different data types (the condition type can refer to Figure 4).

**Figure 6:**
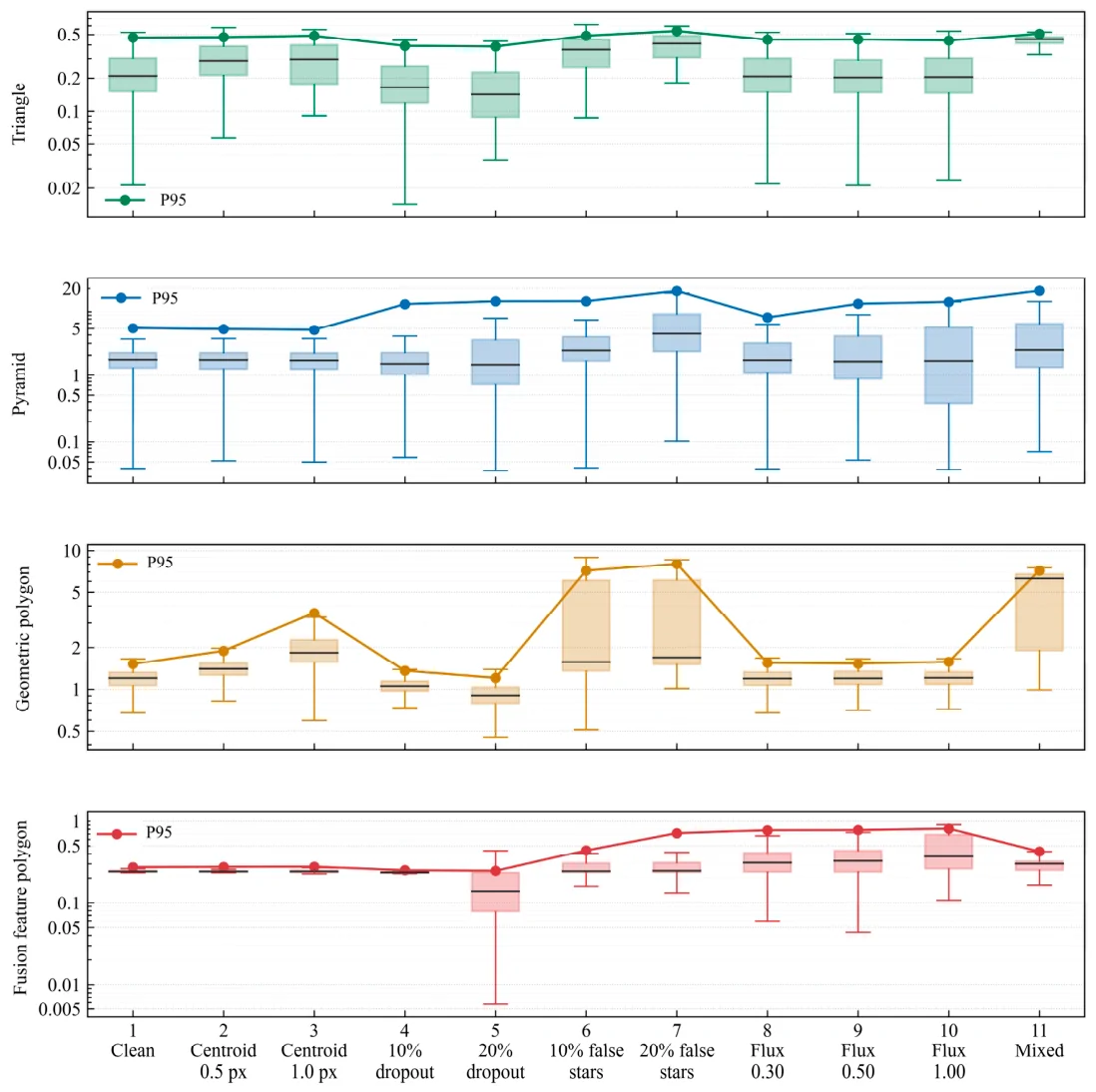
Boxplot distributions of successful-frame solution runtimes for four algorithms in eleven data environments. The black horizontal lines inside the boxes indicate the median (50th percentile) runtime.

**Figure 7:**
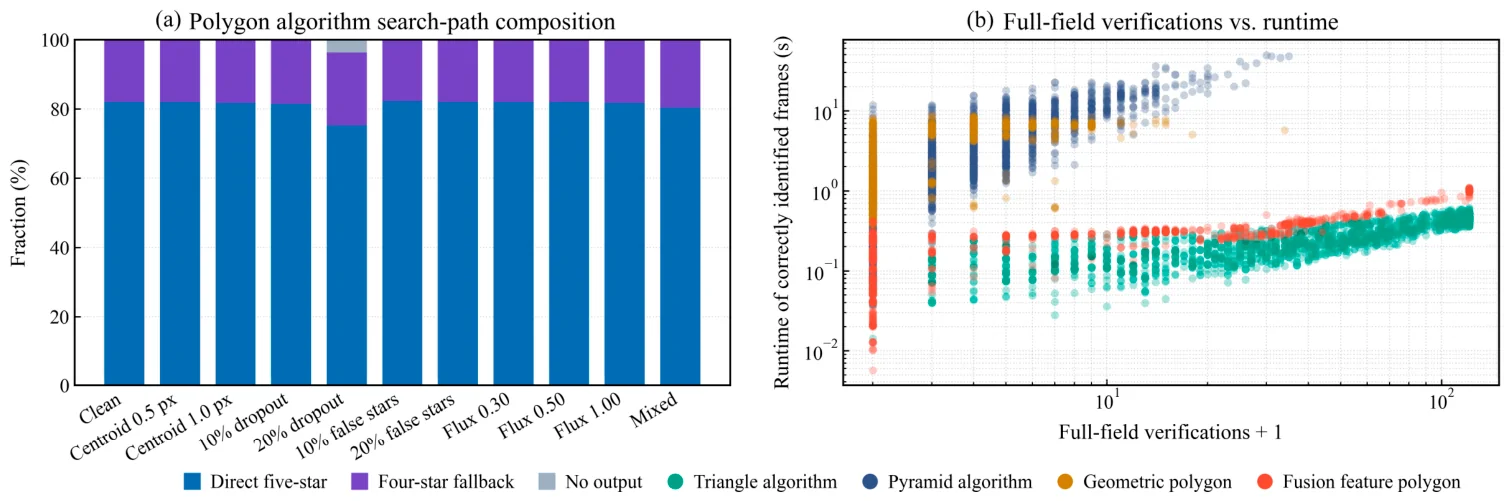
Search-path composition of the Fusion feature polygon algorithm and frame-wise relationships between full-field verification counts and solution runtimes for four algorithms. (**a**) Search-path composition of the fusion feature polygon algorithm under different test conditions. (**b**) Full-field verification count versus runtime for correctly identified frames of the four algorithms.

**Figure 8:**
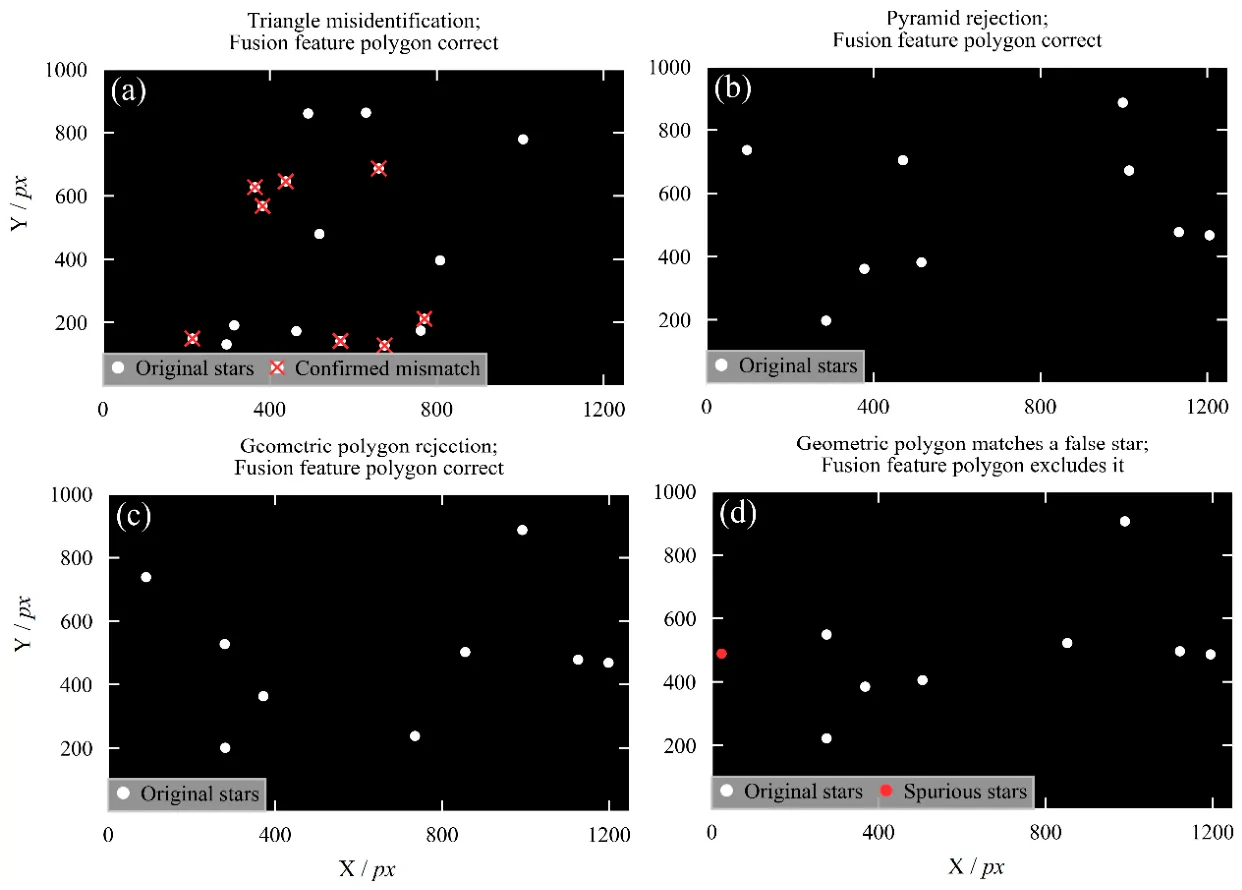
Comparison of identification results for four representative frame types. (**a**) Triangle misidentification versus correct identification by the proposed algorithm. (**b**) Pyramid rejection versus correct identification by the proposed algorithm. (**c**) Geometric polygon rejection versus correct identification by the proposed algorithm. (**d**) False-star matching by the geometric polygon algorithm versus false-star exclusion by the proposed algorithm.White dots indicate genuine observed stars, red dots indicate injected false stars, and red crosses indicate confirmed individual-star mismatches.

**Table 1:** Parameter settings of the Fusion feature polygon algorithm.

Stage	Parameter	Symbol	Value
Matching decision	Decision parameter	α	$1.0\times 10^{-3}$
Matching decision	Five-star decision threshold	$\tau_{5}^{2}$	$\tau_{5}^{2}=\chi_{17}^{2}(1-\alpha )=\chi_{17}^{2}(0.999)=40.7902$
Matching decision	Four-star decision threshold	$\tau_{4}^{2}$	$\tau_{4}^{2}=\chi_{12}^{2}(1-\alpha )=\chi_{12}^{2}(0.999)=32.9095$
Candidate retrieval	k-d-tree neighbor count	k	k=400 The candidate set is {50,100,200,400}
Candidate retrieval	Maximum angular distance for neighbor selection	$d_{\max}$	14.2° (constrained by the field of view)
Fallback and verification	Fifth-star consistency threshold	$\tau_{v}$	0.12°
Fallback and verification	Maximum number of reference-star changes	$N_{\mathrm{ref}\_\mathrm{switch}}$	120
Fallback and verification	Maximum number of full-field verifications	$N_{\mathrm{verify},\max}$	120
Correctness assessment	Frame-wise assessment criterion	$\varepsilon_{b}$	≤0.2°

**Table 2:** Identification rates and misidentification counts of four algorithms in eleven data environments.

Condition	*N*	Triangle		Pyramid		Geometric Polygon		Fusion Feature Polygon	
		Identification Rate/%	Misidentified Frames	Identification Rate/%	Misidentified Frames	Identification Rate/%	Misidentified Frames	Identification Rate/%	Misidentified Frames
Unperturbed data	372	66.67	12	84.95	0	81.45	0	**92.74**	0
Centroid noise $\sigma_{p}$ = 0.5 px	372	31.18	27	84.68	0	81.18	0	**92.20**	0
Centroid noise $\sigma_{p}$ = 1.0 px	372	14.78	55	84.68	0	80.65	0	**92.20**	0
10% star dropout	372	67.47	10	84.14	0	62.37	0	**88.98**	1
20% star dropout	372	62.63	5	80.11	0	40.32	0	**86.02**	0
10% false stars	372	58.60	28	84.68	0	81.18	0	**92.20**	0
20% false stars	372	54.03	49	83.87	0	80.65	0	**92.20**	3
Photometric perturbation $\sigma_{F}$ = 0.3	372	66.67	12	**84.95**	0	81.45	0	82.80	0
Photometric perturbation $\sigma_{F}$ = 0.5	372	66.67	12	**84.95**	0	81.45	0	81.18	0
Photometric perturbation $\sigma_{F}$ = 1	372	66.67	12	**84.95**	0	81.45	0	77.96	0
Combined perturbations	372	25.00	35	81.99	0	61.83	0	**84.14**	2

Note: *N* is the number of valid frames for each condition; the identification rate uses *N* as the denominator; misidentifications are frames for which the algorithm outputs an incorrect solution; bold values indicate the highest identification rate for each condition.

## Data Availability

The data presented in this study are available on request from the corresponding author.
